# Evaluation of the impact of Covid-19 on air traffic volume in Turkish airspace using artificial neural networks and time series

**DOI:** 10.1038/s41598-023-33784-x

**Published:** 2023-04-21

**Authors:** Nurullah Gultekin, Sibel Acik Kemaloglu

**Affiliations:** 1 Graduate School of Natural and Applied Science, Ankara University, Ankara, Turkey; 2 Department of Statistics, Faculty of Sciences, Ankara University, Ankara, Turkey

**Keywords:** Applied mathematics, Statistics

## Abstract

In early 2020, the aviation sector was one of the business lines adversely affected by the Covid 19 outbreak that affected the whole world. As a result, some countries imposed travel restrictions. Following these restrictions, air traffic density has decreased significantly worldwide. Since air traffic density directly affects almost all operations in air transportation, analyzing these data is very essential. For this purpose, SARIMA models, one of the linear time series models, and multilayer perceptron model (MLP), one of the artificial neural network methods suitable for nonlinear modeling, were applied to the air traffic data regarding Turkish airspace between 2010 and 2019, and the actual air traffic density was compared with the forecasts obtained from these analyses. It is considered that the results of this study are essential for organizations conducting aviation operations to take into consideration while doing future planning.

## Introduction

The World Health Organization (WHO) declared a global pandemic after a new type of contagious Coronavirus that emerged at the end of 2019 showed its effect worldwide at the beginning of 2020^1^. To prevent the spread of the rapidly contagious virus, states have imposed strict travel bans. One of the sectors most affected by these measures was the airline transportation sector. Due to the pandemic, companies withdrew most of the aircraft in their fleets from flights, leading to dramatic reductions in air traffic.

Aviation is a constantly growing industry in the modern world. Therefore, analyzing aeronautical data and making future forecasts is essential for the future plans of all aviation stakeholders. In the document “Manual on Air Traffic Forecasting” published by ICAO (International Civil Aviation Organization), future forecasts have been described using ARIMA models, which are also used and recommended by EUROCONTROL (European Organization for the Safety of Air Navigation). Postorino^2^, Inglada and Rey^3^, Lai and Lu^4^ and Andreoni and Postorino^5^ worked with time series, and ARIMA models. Dingari et al. ^6^ compared ARIMA and Holt-Winters methods for forecasts of domestic flights of 2019 in India. Phyoe et al.^7^ forecasted the long-term air traffic demand in Singapore airspace using time series analysis, and then Phyoe et al.^8^ continued this study with the impact of air traffic on GDP. Chai^9^ used seasonal ARIMA (SARIMA) models to forecast Hong Kong air traffic numbers. Jungmittag^10^ applied SARIMA models to forecast air travel demand at Frankfurt Airport. Dantas et al.^11^ proposed a novel approach (Bootstrap aggregating Holt-Winters method) to forecast air transportation demand. Asrah et al.^12^ used time series to analyze the number of Malaysia Airlines and AirAsia Airlines passengers. Gudmundsson et al.^13^ estimated the relationship between the strength of economic shocks and temporal recovery in the world air transport industry in terms of Covid 19. La et al.^14^ compared 3 different forecasting methods (exponential smoothing, ARIMA, and gray forecasting method) to predict the passenger traffic volume of civil aviation in China. Al-Sultan et al.^15^ compared multiple forecasting methods to predict Kuwait air passenger data. Deng^16^ discussed the development of China’s air transportation and examined the impact of the Covid 19 on the airline industry. Borucka et al.^17^ identified the time series describing the number of airline flights in Poland in the context of the Covid-19 pandemic. Tolcha^18^ estimated the immediate and long-term effects of COVID-19 on air transport markets and forecasted timescales for the recovery of the markets for domestic and international flights in nine African countries using the SARIMAX method.

Artificial neural networks (ANNs) method is one of the most widely used methods for forecasting the future. It is an analysis method inspired by the learning ability of the human brain and the biological nervous system. In this method, the computer system learns the relationships between events from examples and makes decisions about subsequent examples^19^. One of the most important features of neural network analysis is that it is suitable for nonlinear modeling by making no assumptions about the distribution of observations^20^.

Busquets et al.^21^ used artificial neural networks to forecast air traffic numbers in the United States and Saadaoui et al.^22^ used hybrid artificial neural networks for a similar purpose. Cicek and Ozturk^23^ included airline passenger data in their study while comparing hybrid models of artificial neural networks with support vector regression and ARIMA models taking into account different data sources. Weatherford et al.^24^ compared traditional forecasting methods with neural network methods on 85-week airline data. Blinova^25^ used the artificial neural networks method to make forecasts for airline passenger flow in Russian airspace between 2006 and 2010. Srisaeng et al.^26^ used artificial neural networks to forecast Australian domestic passenger demand. Makridakis et al.^27^ compared traditional statistical and machine learning methods in terms of time series forecasting. Zheng et al.^28^ investigated both parametric and nonparametric machine learning models in the air transportation industry in the United States. Jafari^29^ investigated COVID-19’s impact on the US domestic air passenger demand and used time series and neural network forecasting methods. Januschowski et al.^30^ discussed the spectrum of statistical and machine learning methods in terms of forecasting.

In this study, the impact of the global pandemic on air traffic density has been analyzed, based on the example of Turkish airspace. Monthly total air traffic data (domestic, international, and overflight) between 2010 and 2019 has been taken into account. First, SARIMA (Seasonal Auto-Regressive Integrated Moving Average), one of the linear forecasting methods, has been applied to obtain forecasts for 2020–2024. Later, multi-layer perceptron (MLP), one of the artificial neural network methods, has been used to obtain the forecast for the same period. Afterward, the MLP forecast that showed better performance was compared with the actual air traffic data, and the impact of the Covid 19 pandemic on air traffic density was attempted to be revealed.

## Materials and methods

### Data description

Aviation activities include many elements such as civil and military activities. ICAO classifies air services into two main categories for statistical purposes: commercial air transport and general aviation. Commercial air transport is the operation of aircraft on one or more stages on a scheduled or non-scheduled basis, which is available to the public for remuneration and hire. General aviation is all civilian operations other than scheduled air services and non-scheduled air transport operations for remuneration or hire (such as business flights, aerial work, instructional and pleasure flights)^31^. In this study, all monthly civil and military flights that used Turkish airspace between 2010 and 2019 were considered for the purpose of reflecting the air traffic density. Because not only commercial air transportation but also some part of general aviation activities reflects the air traffic density. The data were extracted from DHMI’s (air navigation service provider of Turkey) website^32^.

### Box-Jenkins method

The Box-Jenkins method is a time series analysis and forecasting technique that was developed by Box and Jenkins in the 1970s. The method involves a series of steps for identifying, estimating, and diagnosing a suitable autoregressive integrated moving average (ARIMA) model for a given time series data. The ARIMA model is visualized as $ARIMA(p,d,q),$ in which $p$ represents the order of the autoregressive (AR) model, $q$ represents the order of the moving average (MA) model, and $d$ represents the order of differencing.

The SARIMA (Seasonal Autoregressive Integrated Moving Average) model is an extension of the ARIMA model that takes into account seasonal patterns in the data. ARIMA models are used to model non-stationary time series data by differencing the data to make it stationary and then modeling the resulting stationary series using autoregressive (AR), integrated (I), and moving average (MA) terms. The SARIMA model adds a seasonal component to this, which captures the pattern that repeats itself over a fixed time interval, such as a day, week, or year.

The SARIMA model is denoted as $SARIMA(p,d,q){\left(P,D,Q\right)}_{s}$ , and mathematical form of the model is given as follows:

$${\Phi }_{P}\left({L}^{s}\right){\varphi }_{p}\left(L\right){\left(1-L\right)}^{d}{\left(1-{L}^{s}\right)}^{D}{y}_{t}={\Theta }_{Q}({L}^{s}){\theta }_{q}(L){e}_{t}$$

where $p$ is autoregressive term (AR), $d$ is integrated term, $q$ is moving average term (MA), $P$ is seasonal autoregressive term (SAR), $D$ is seasonal integrated term, $Q$ is seasonal moving average term (SMA), $s$ is the number of periods in each season, $L$ is back-shift operation as $L{y}_{t}={y}_{t-1}$*,* ${e}_{t}$ is white noise ($WN(0,{\sigma }^{2})$, ${\left(1-L\right)}^{d}$ is non-seasonal, and ${\left(1-{L}^{s}\right)}^{D}$ is seasonal differencing operator.

Box and Jenkins proposed a 3-step iterative approach to SARIMA model selection in accordance with the principle of model simplicity. These are model identification, parameter estimation, and diagnostic check. These 3 steps are repeated until a satisfactory model is found. The selected model is then used for future forecasting of the series^33^.

### Artificial neural networks (ANNs) and multilayer perceptron models (MLP)

Although it first emerged to be used in studies in the field of biology, artificial neural networks (ANNs) is a machine learning technique that is frequently used today and became the subject of studies in different fields. One of the essential features of ANNs is that it is suitable for nonlinear modeling by making no assumptions about the distribution of observations. In other words, ANNs models do not need to assume probability distribution for the data along with formal requirements and can therefore be characterized as versatile^34^.

While performing analysis with ANNs, the computer system imitates the learning way of the human brain, notices and experiences specific patterns related to the data in hand, and makes inferences about the data^19^. An ANN is generally expressed as three layers (input, hidden, output). Each layer contains essential processing elements. These elements are commonly called nodes or neurons in the literature^35,36^.

The MLP model, on the other hand, is a characterized form of ANNs consisting of one or more hidden layers^20^. The MLP model has an input, an output, and one or more hidden layers where each node is irreversibly dependent on each other. The relationship between the inputs, ${y}_{t-i}(i=\mathrm{1,2},\dots ,p)$ and the outputs ${y}_{i}$ can be represented by the following formula representing the hidden nodes *p* and* h* in the MLP model as:

$${y}_{t}=G\left({\alpha }_{0}+\sum_{j=1}^{h}{\alpha }_{j}F\left({\beta }_{0j}+\sum_{i=1}^{p}{\beta }_{ij}{y}_{(t-i)}\right)\right)$$

where ${\alpha }_{j}; {\beta }_{ij}\left(i=\mathrm{1,2},\dots ,p;j=\mathrm{1,2},\dots ,h\right):$ link weight, ${\alpha }_{0}; {\beta }_{0j}$∶ bias term, *F*: hidden layer activation function, *G: *output layer activation function.

**Figure 1:**
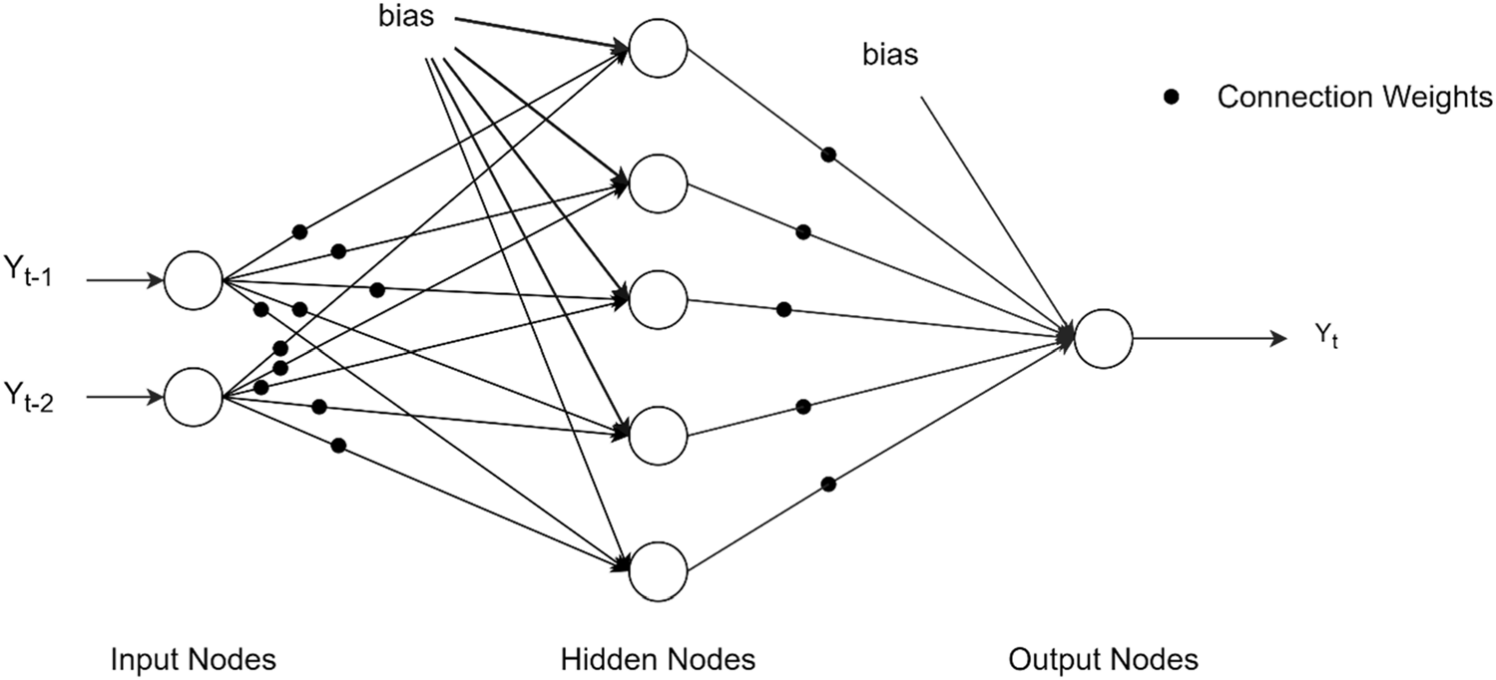
A MLP network for one-step-ahead forecasting based on two lagged terms.

The MLP model uses Back Propagation (BP), which is a tool that is used to improve forecast accuracy in machine learning and data mining. In an artificial neural network, after the input data is propagated to the decision point with the network parameters, the error is back-propagated, and the parameters are updated. Thus, back propagation takes the error associated with a wrong guess by a neural network and uses that error to adjust the neural network parameters toward less error^37^. An MLP network for one-step-ahead forecasting based on two lagged terms has been shown in Fig. 1.

### Model selection and performance criteria

One of the most critical steps in the model selection is to determine the most appropriate model. The Akaike Information Criterion (AIC) is a commonly used model selection criterion. It is calculated mathematically with the following formula:

$${AIC}_{(p)}=n ln\left(\frac{{\sigma }_{e}^{2}}{n}\right)+2p$$

where; *n*: the number of observations, ${\sigma }_{e}^{2}$: the sum of squares of sample residuals.* p*: the number of parameters in the model.

The model that has the smallest AIC is taken as the optimum model. In the time series analysis, there are different measurement criteria to measure the performance of the forecasts made by the selected models. In this study, RMSE (Root Mean Squared Error) and MAPE (Mean Absolute Percentage Error) are used. RMSE is a measure of the squared deviation from the average and gives general information about the error. MAPE is a measure representing the percentage of average absolute error that occurred.

The mathematical formulas for RMSE and MAPE are given as follows:

$$RMSE=\sqrt{MSE}=\sqrt{\frac{1}{n}\sum_{t=1}^{n}{e}_{t}^{2}} \quad MAPE=\frac{1}{n}\sum_{t=1}^{n}\left|\frac{{e}_{t}}{{y}_{t}}\right|\times 100,$$

where; *n*: the number of observations, ${e}_{t}$: errors, ${y}_{t}$: observation values.

## Application

**Figure 2:**
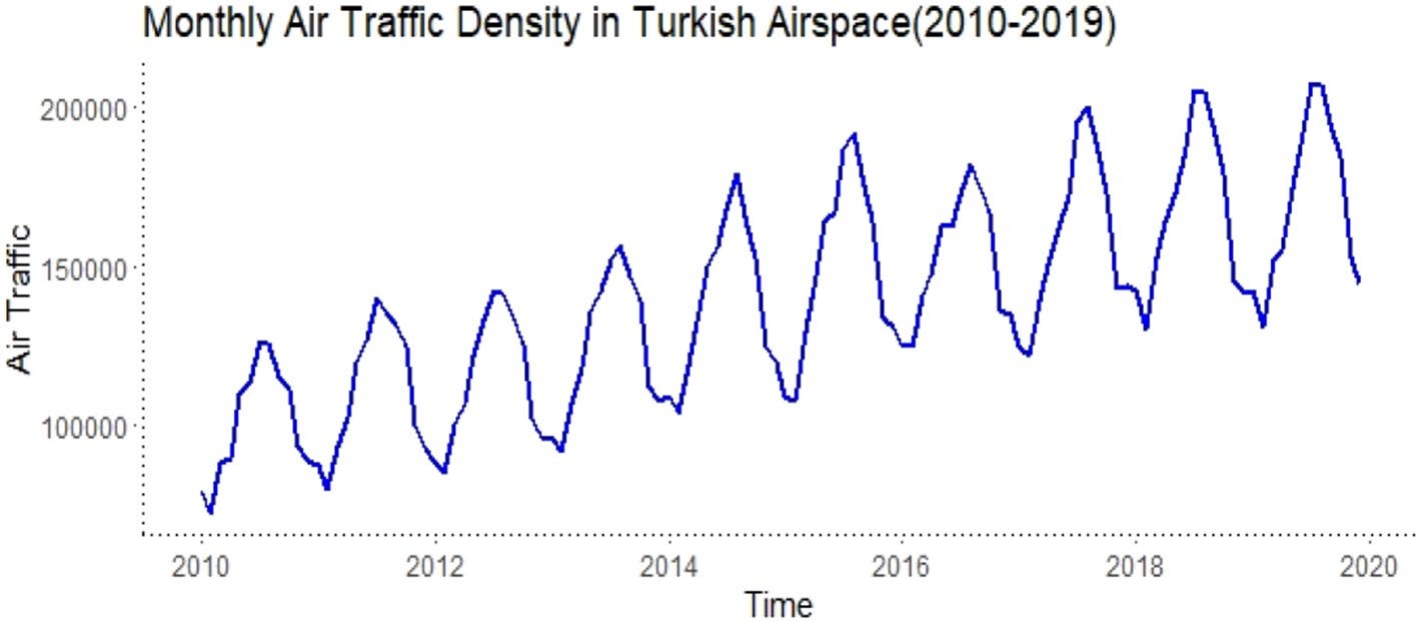
Graphical representation of monthly air traffic data for the years 2010–2019.

The data are numerically shown in Appendix A1. Descriptive statistics of the data are presented in Table 1, and the relevant data are visually represented by year, as shown in Fig. 2.

**Table 1:** Descriptive statistics on monthly air traffic data using Turkish airspace between 2010 and 2019.

Year	Min	1st Quantile	Median	Mean	3rd Quantile	Max
2010	72,392	88,978	101,392	101,094	114,038	126,279
2011	79,986	93,220	110,991	111,265	128,538	139,851
2012	84,948	98,988	114,224	114,707	133,255	142,566
2013	91,602	107,377	126,773	125,414	143,652	156,455
2014	103,867	119,505	141,260	139,914	158,281	179,080
2015	108,024	130,523	153,571	150,408	168,987	192,220
2016	124,678	136,072	155,206	152,492	168,160	182,014
2017	122,214	142,176	156,462	159,501	176,159	200,388
2018	130,025	144,444	168,695	168,102	188,216	205,165
2019	130,894	150,259	164,897	169,536	189,909	207,678
2010–2019	72,392	114,599	139,066	139,243	163,178	207,678

**Figure 3:**
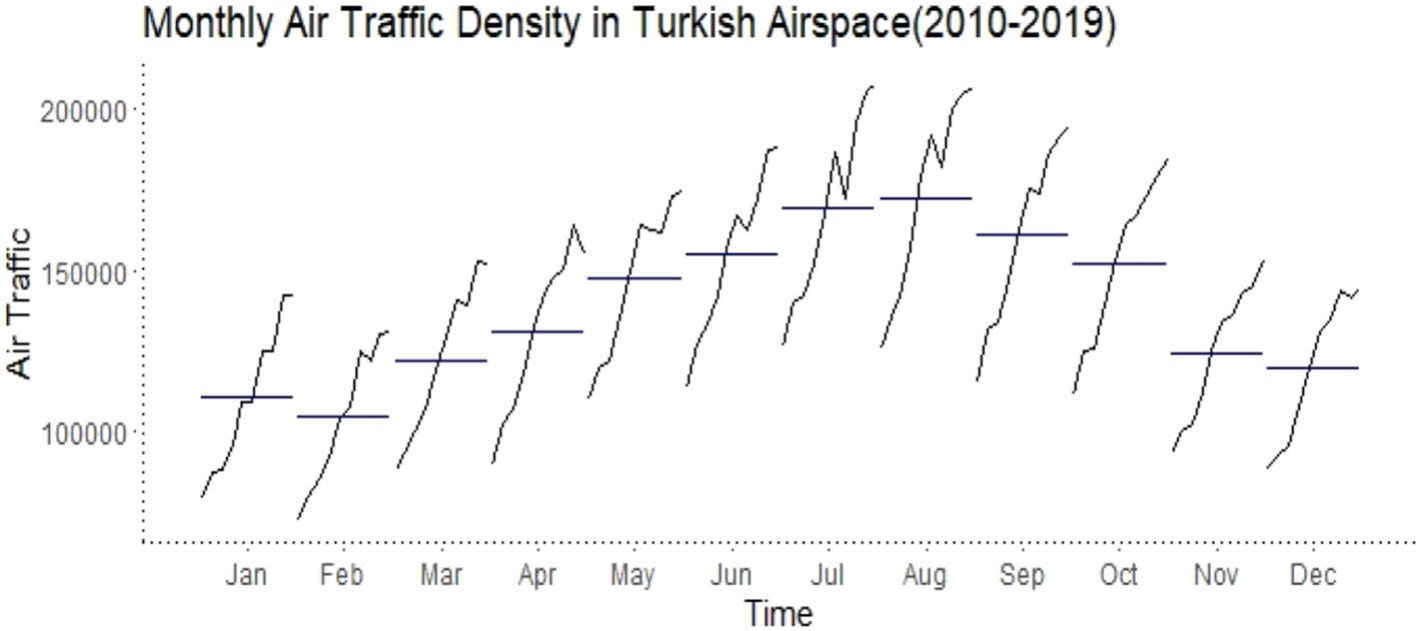
Monthly averages of air traffic data for the years 2010–2019.

In Fig. 2, it can be noticed that the series shows an increase over the years (i.e. it has a trend) and shows regular increases, especially in certain months. This gives the impression that there is seasonality in the series. To see this better, we can look at Fig. 3, which shows the average of each month of the series.

A closer examination of Figs. 2 and 3 reveals that air traffic density in Turkish airspace peaks in the summer, while it decreases in the winter. Moreover, it is observed that the fastest increase acceleration was experienced between 2013 and 2016. In the summer of 2016, a conjuncture-driven decline was particularly noticeable.

**Figure 4:**
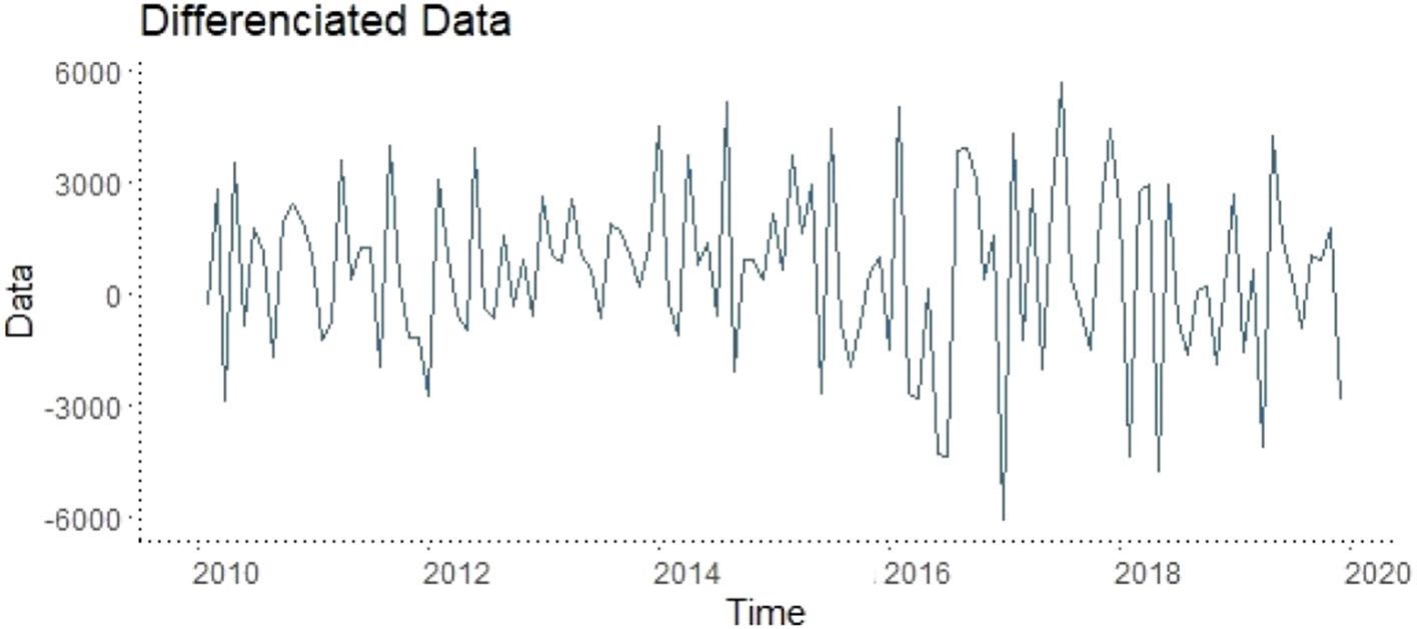
Graphical representation of the series of air traffic data for the years 2010–2019 after applying the differentiation process.

After all these inferences, the relevant data were firstly seasonally adjusted using the tramo/seats approach. Then, a one-time differencing process was applied to make the data stationary and the series is shown in Fig. 4.

Subsequently, many model tests were conducted for the related series, and AIC, RMSE and MAPE values are presented in Table 2.

**Table 2:** Comparison of the performances of different SARIMA model trials.

Model	AIC	RMSE	MAPE
SARIMA(1,0,0)(1,1,1)_12_	− 441.24	3789.088	2.113225
SARIMA(0,1,1)(0,1,1)_12_	− 444.56	3993.933	2.106652
**SARIMA(1,1,1)(1,1,1)**_**12**_	− **446.33**	**3611.427**	**1.869622**
SARIMA(1,1,1)(0,1,1)_12_	− 446.07	3915.209	2.041757
SARIMA(1,0,0)(0,1,1)_12_	− 440.40	4053.558	2.244071
SARIMA(0,1,2)(0,1,1)_12_	− 444.51	3955.186	2.079091
SARIMA(1,1,2)(1,1,1)_12_	− 444.44	3613.576	1.872375

Significant values are in bold.

**Figure 5:**
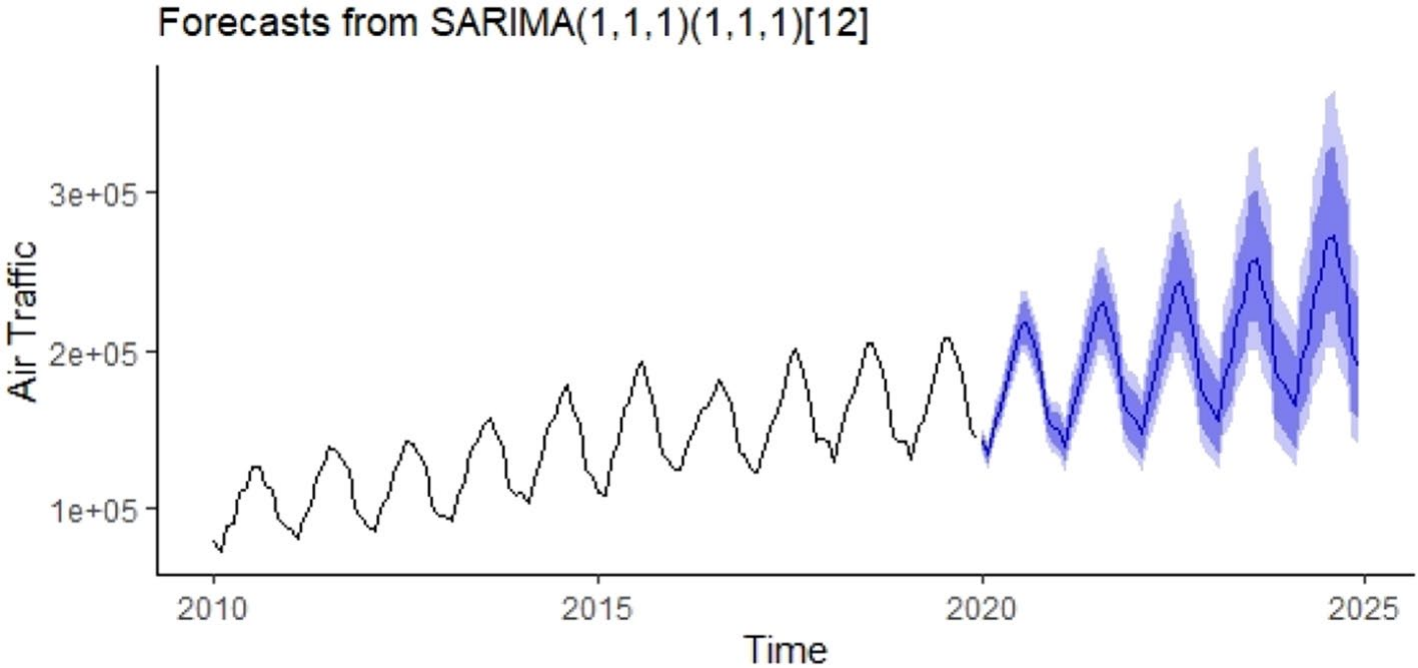
Forecasts for the years 2020–2024 with the help of the SARIMA(1,1,1)(1,1,1)_12_ model.

As can be seen from Table 2, the most appropriate model was determined as SARIMA (1,1,1)(1,1,1)_12_ and forecasts for the years 2020–2024 were calculated with the help of this model and shown in Fig. 5. The obtained forecasts are presented numerically in Appendix A2.

After these analyses and forecasts made with the SARIMA model, an MLP model suitable for the data presented in Table 1 was determined, and forecasts for the years 2020–2024 were obtained.

**Figure 6:**
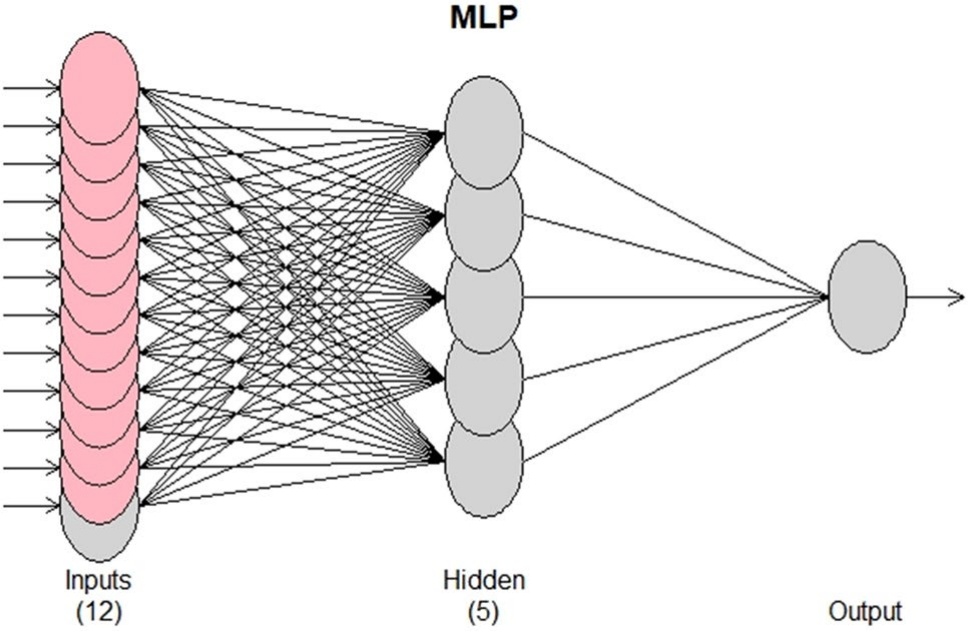
MLP model.

There is no traditional method in the literature to determine the most appropriate neural network model for a dataset. However, methods such as iterative neural filters have been proposed to select the most appropriate model among many^8^. Based on this fact, the visualized version of the MLP model is shown in Fig. 6. As seen in Fig. 6, the model consists of 1 hidden layer and 5 nodes in the hidden layer. There were 12 nodes in the input layer. Lagged terms are modeled separately as 2, 3, 4, 5, 6, 7, and 12 respectively and the model performances are found according to RMSE and MAPE values, as shown in Table 3.

**Table 3:** Comparison of the performances of different MLP model trials.

Model	RMSE	MAPE
MLP (lagged terms = 2)	3566.896	2.059840
MLP (lagged terms = 3)	5497.596	2.523029
MLP (lagged terms = 4)	3615.040	1.928299
**MLP (lagged terms = 5)**	**2744.905**	**1.652927**
MLP (lagged terms = 6)	4029.886	2.000589
MLP (lagged terms = 7)	3652.395	1.975998
MLP (lagged terms = 12)	3574.459	2.193626

Significant values are in bold.

**Figure 7:**
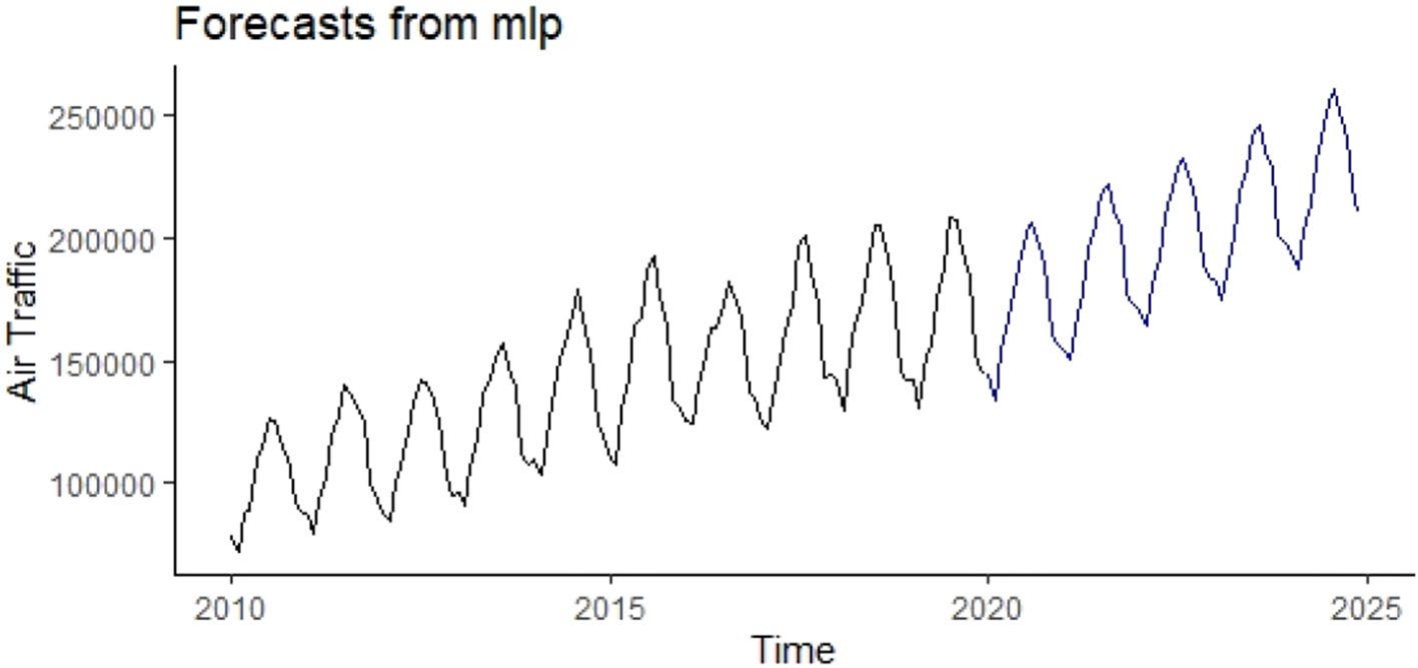
Forecasts obtained using the MLP model.

From Table 3, it can be seen that the MLP model that has 5 lagged terms showed the best performance according to RMSE and MAPE. Using the aforementioned MLP model, the calculated forecasts for the years between 2020 and 2024 were presented in Fig. 7 and Appendix A3. When Fig. 7 is examined, it draws the attention that the forecasts obtained with the help of the MLP model preserve the seasonal fluctuations and trends of the past period.

SARIMA and MLP model performances were compared by taking into consideration each method's RMSE and MAPE criteria, as shown in Table 4. It is understood that the forecasts obtained with the help of the MLP model yielded better results than the SARIMA (1,1,1)(1,1,1)_12_ model with respect of RMSE and MAPE values of both methods.

**Table 4:** Comparing the performances of the SARIMA and MLP models.

Model	RMSE	MAPE
$SARIMA(\mathrm{1,1},1){(\mathrm{1,1},1)}_{12}$	3611.427	1.869622
$MLP(lags=5)$	2744.905	1.652927

## Conclusions

**Figure 8:**
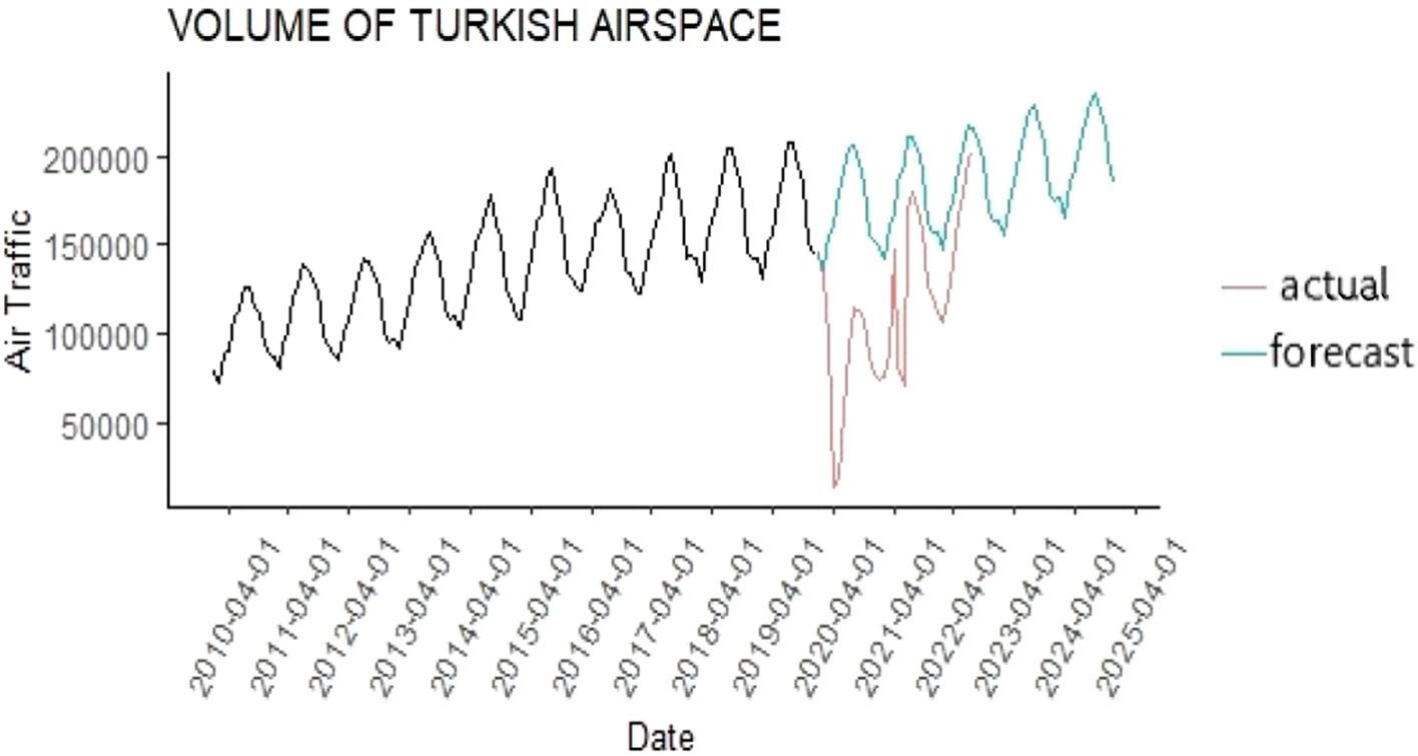
Comparison of the forecasts obtained with the help of the MLP model and the actual air traffic density.

For the organizations responsible for managing air traffic, it was beneficial to compare the future forecasts obtained in this study with the actual air traffic density. Analyzing the effects of the global pandemic is expected to help in comprehend the negativities it caused and take lessons from it. Also, it is considered beneficial to shape the future of aviation. In Fig. 8, the course of air traffic density between 2010 and 2019 and the forecasts for the years 2020–2024 are presented. Additionally, air traffic density until 2022 is also included in this display.

It is seen from Fig. 8 that air traffic density has an increasing trend in Turkey. However, from the beginning of 2020, the travel restrictions imposed by the states due to the global Covid 19 pandemic, have adversely affected air traffic density. Especially in April 2020, air traffic density reached its lowest point. The easing of travel restrictions in the summer of 2021 has caused air traffic density to increase again.

This study investigates the impact of the Covid 19 pandemic on the air traffic density in Turkey. The data were obtained directly from the air navigation service provider of Turkey (DHMI) website in raw format. All the data were extracted manually from pdf-formatted files. It is not surprising to see that air traffic density and travel restrictions have a strong negative correlation. But it can be seen from Fig. 8 that as soon as travel restrictions are eased, the demand for air transportation increases rapidly. This is evidence of the importance of air transportation in the modern world.

Planning is very crucial for organizations conducting aviation activities. It is essential to predict future demand so that all related activities can be planned accordingly. This study represented the forecasts obtained from the SARIMA and MLP models and compared their performances. The MLP model yielded better results. Later, the forecasts obtained from the MLP model were compared to actual traffic density to determine the magnitude of the pandemic effect on air traffic in Turkey.

Another important conclusion is that air transportation became indispensable in the modern world. When there are no restraints, air transportation demand constantly grows.

Further analysis can be done with other data sources in aviation such as domestic flights, international flights, passenger demand, etc. With more observed data, better modeling and comparison can be performed easily.

## Supplementary Information

Supplementary Information.

## Data Availability

The dataset used and/or analyzed during the current study was available from the corresponding author upon reasonable request.
