# Supplementary material for: Evaluation of the impact of Covid-19 on air traffic volume in Turkish airspace using artificial neural networks and time series
Source: Sci Rep. 2023 Apr 21;13:6551. doi: 10.1038/s41598-023-33784-x (PMC10119839; doi:10.1038/s41598-023-33784-x)
Supplement: Supplementary file 1 — Supplementary Information. [file 41598_2023_33784_MOESM1_ESM.docx]

**Appendix**

**Appendix A1**

Monthly air traffic numbers using Turkish airspace between 2010-2019 (data)

|  | **2010** | **2011** | **2012** | **2013** | **2014** | **2015** | **2016** | **2017** | **2018** | **2019** |
| --- | --- | --- | --- | --- | --- | --- | --- | --- | --- | --- |
| **Jan** | 78896 | 87196 | 87904 | 95922 | 109538 | 109538 | 125318 | 124678 | 142422 | 142339 |
| **Feb** | 72392 | 79986 | 84948 | 91602 | 103867 | 108024 | 124678 | 122214 | 130025 | 130894 |
| **Mar** | 88262 | 93331 | 100070 | 106909 | 118690 | 129606 | 140506 | 139054 | 152531 | 152144 |
| **Apr** | 89519 | 102203 | 106752 | 118079 | 132378 | 143080 | 147624 | 150844 | 164083 | 155351 |
| **May** | 109725 | 119779 | 121696 | 135467 | 150141 | 164359 | 162787 | 162080 | 173306 | 174443 |
| **Jun** | 113758 | 127506 | 132942 | 142495 | 156857 | 166806 | 162883 | 172397 | 187250 | 188273 |
| **Jul** | 126279 | 139851 | 142566 | 151836 | 168075 | 186745 | 172181 | 196068 | 205165 | 207678 |
| **Aug** | 125726 | 135652 | 141398 | 156455 | 179080 | 192220 | 182014 | 200388 | 204912 | 207237 |
| **Sep** | 114879 | 131634 | 134193 | 147123 | 162551 | 175530 | 173864 | 186057 | 191112 | 194817 |
| **Oct** | 111414 | 124961 | 125848 | 139077 | 153224 | 164061 | 166820 | 172860 | 179438 | 184129 |
| **Nov** | 93058 | 100199 | 102428 | 112475 | 124793 | 134096 | 136528 | 143217 | 145118 | 152522 |
| **Dec** | 89217 | 92887 | 95741 | 107533 | 119777 | 130828 | 134705 | 144160 | 141858 | 144603 |

**Appendix A2**

Forecasts calculated for 2020-2024 with SARIMA (1,1,1)(1,1,1)_12_ model

|  | **2020** | **2021** | **2022** | **2023** | **2024** |
| --- | --- | --- | --- | --- | --- |
| **Jan** | 142708.6 | 148327.7 | 155956.7 | 164739.6 | 174341.8 |
| **Feb** | 133628.4 | 139727.3 | 147269.2 | 155714.1 | 164854.5 |
| **Mar** | 156042.0 | 163349.3 | 172244.7 | 182155.1 | 192861.9 |
| **Apr** | 163560.4 | 172961.2 | 183123.5 | 193976.8 | 205513.5 |
| **May** | 185020.6 | 196189.1 | 207943.9 | 220365.4 | 233513.0 |
| **Jun** | 196814.9 | 207454.0 | 219356.9 | 232236.1 | 245996.5 |
| **Jul** | 216080.4 | 227312.4 | 240164.3 | 254184.1 | 269210.4 |
| **Aug** | 217603.3 | 229747.8 | 243092.2 | 257434.1 | 272716.9 |
| **Sep** | 203762.8 | 214791.0 | 227120.5 | 240457.9 | 254706.5 |
| **Oct** | 192756.3 | 203258.8 | 214956.1 | 227591.9 | 241083.4 |
| **Nov** | 158599.5 | 166788.6 | 176195.1 | 186470.6 | 197489.8 |
| **Dec** | 151633.9 | 160002.2 | 169255.4 | 179224.0 | 189856.6 |

**Appendix A3**

Forecasts calculated for 2020-2024 with the MLP model

|  | **2020** | **2021** | **2022** | **2023** | **2024** |
| --- | --- | --- | --- | --- | --- |
| **Jan** | 143542.4 | 155640.2 | 169826.5 | 184261.0 | 196312.4 |
| **Feb** | 133596.1 | 150140.2 | 164820.1 | 175708.8 | 188380.3 |
| **Mar** | 152591.0 | 167945.7 | 181401.5 | 192262.3 | 204803.2 |
| **Apr** | 161047.2 | 178381.3 | 192541.3 | 203082.8 | 215226.4 |
| **May** | 178318.0 | 194716.7 | 210119.0 | 220757.6 | 232912.4 |
| **Jun** | 188568.3 | 204350.9 | 218514.1 | 229893.4 | 241924.9 |
| **Jul** | 202591.8 | 217835.0 | 231456.1 | 243476.0 | 255033.3 |
| **Aug** | 206668.8 | 222382.3 | 236025.5 | 248181.9 | 259884.8 |
| **Sep** | 197605.6 | 212047.2 | 224840.6 | 236775.1 | 249105.9 |
| **Oct** | 189273.5 | 203840.2 | 216905.3 | 228751.7 | 241320.2 |
| **Nov** | 162070.9 | 177139.5 | 190281.2 | 203172.8 | 214136.6 |
| **Dec** | 157781.1 | 173404.8 | 186388.5 | 199172.1 | 210451.0 |
